# Suppression of Phospholipase Dγs Confers Increased Aluminum Resistance in *Arabidopsis thaliana*

**DOI:** 10.1371/journal.pone.0028086

**Published:** 2011-12-07

**Authors:** Jian Zhao, Cunxi Wang, Mohamed Bedair, Ruth Welti, Lloyd W. Sumner, Ivan Baxter, Xuemin Wang

**Affiliations:** 1 Department of Biochemistry, Kansas State University, Manhattan, Kansas, United States of America; 2 Division of Biology, Kansas State University, Manhattan, Kansas, United States of America; 3 Plant Biology Division, The Samuel Roberts Noble Foundation, Ardmore, Oklahoma, United States of America; 4 USDA-ARS Plant Genetics Research Unit, St. Louis, Missouri, United States of America; 5 Donald Danforth Plant Sciences Center, St. Louis, Missouri, United States of America; 6 Department of Biology, University of Missouri, St. Louis, Missouri, United States of America; Korea University, Republic of Korea

## Abstract

Aluminum (Al) toxicity is the major stress in acidic soil that comprises about 50% of the world's arable land. The complex molecular mechanisms of Al toxicity have yet to be fully determined. As a barrier to Al entrance, plant cell membranes play essential roles in plant interaction with Al, and lipid composition and membrane integrity change significantly under Al stress. Here, we show that phospholipase Dγ*s* (PLDγ*s*) are induced by Al stress and contribute to Al-induced membrane lipid alterations. RNAi suppression of *PLD*γ resulted in a decrease in both *PLD*γ*1* and *PLD*γ*2* expression and an increase in Al resistance. Genetic disruption of *PLD*γ*1* also led to an increased tolerance to Al while knockout of *PLD*γ*2* did not. Both RNAi-suppressed and *pld*γ*1-1* mutants displayed better root growth than wild-type under Al stress conditions, and *PLD*γ*1-*deficient plants had less accumulation of callose, less oxidative damage, and less lipid peroxidation compared to wild-type plants. Most phospholipids and glycolipids were altered in response to Al treatment of wild-type plants, whereas fewer changes in lipids occurred in response to Al stress in PLDγ mutant lines. Our results suggest that PLDγs play a role in membrane lipid modulation under Al stress and that high activities of PLDγs negatively modulate plant tolerance to Al.

## Introduction

Aluminum (Al) toxicity is the major stress in acidic soil mainly because it increases plant susceptibility to other stresses such as nutrient deficiencies and mineral toxicities [1], [2]. Acid-soil regions comprise more than 50% of the world's arable land, and Al toxicity causes large losses in agricultural production [1], [2]. Multiple mechanisms have been implicated in plant response and resistance to Al stress [1]–[3]. Al exposure rapidly inhibits root elongation and subsequently alters root morphology. Some of Al's effects include alteration of cell wall properties, disruption of membrane integrity, alteration in lipid composition, perturbation of Ca^2+^ homeostasis, dysfunction of mitochondria, denaturation of cellular proteins, damage to DNA, and blockage of cell-cycle progression [1], [4]–[8]. Given the wide range of cellular effects, it is thought that multiple factors, both constitutively expressed and induced, are involved in plant response to Al stress and contribute to Al resistance [2]. The primary line of defense is likely secretion of Al-chelating organic acids including malate or citrate into the rhizosphere by transporters from the Al-activated malate transporter (ALMT) and multidrug and toxin extrusion (MATE) families [9]–[12]. A large number of other genes are likely involved in the response to Al stress, and only a few have been characterized [8].

While the Al stress alters the cell wall, the plasma membrane is the barrier for Al entry into cells and probably the first site of Al damage to the cell. Al stress causes a significant decrease in levels of glycolipids and phospholipids and a loss of membrane integrity [7], [13], [14]. The total amount of lipids in maize roots and shoots decreases under Al stress, although several phospholipids increase, including phosphatidylcholine (PC), phosphatidylinositol (PI), and monogalactosyldiacylglycerol (MGDG) [15]. Under Al stress, the activities of phosphatidylinositol 4,5-bisphosphate (PIP_2_)-specific phospholipase C (PLC) and inositol 1,4,5 trisphosphate (IP_3_) levels decrease whereas those of phosphatidylinositol 4-kinase, phosphatidylinositol phosphate 5-kinase, and diacylglycerol kinase increase, suggesting alterations in levels of signaling phospholipids [16], [17]. In addition, Al stress enhanced the peroxidation of membrane lipids and inactivated membrane proteins because reactive oxygen species accumulated in response to Al stress [6], [18]–[20]. One possible mechanism for Al-induced lipid changes may be that Al binds to negatively-charged lipids, such as PIP_2_, PG, and PI; this binding may alter the ability of proteins to interact with lipids, altering membrane enzyme activities and initiating membrane lipid turnover, or causing changes in membrane permeability and cellular processes [16]. These changes in turn could alter membrane composition and properties, as well as the activity of membrane-integrated transporters [21]–[23].

One potential approach for altering plant responses to Al is changing the membrane lipid composition. Elevation of (8Z)-unsaturated long-chain base levels in plant sphingolipids, by constitutively expressing (8E/Z)-desaturase, reversed Al induced root inhibition [7]. Overexpression of a wheat phosphatidylserine synthase gene (*TaPSS1*) increased PS and PC content in transgenic Arabidopsis and yeast, while deletion of the PSS gene decreased Al resistance in yeast [24]. Al was shown to inhibit the PLC and phospholipase D (PLD) pathways and reduce the formation of phosphatidic acid (PA) in plant tissue cultures [25]–[28]. However, the effect of PLDs on plant tolerance to Al and the identities of the specific PLDs involved remain unknown.

PLD catalyzes the hydrolysis of phospholipids to generate PA and a free head group [29], [30]. PLD is a major family of lipid-hydrolyzing enzymes in plants and 12 PLDs have been identified in Arabidopsis, including 3 PLDγs that are highly homologous. PLDs are activated under various stress conditions and specific PLD family members have been shown to play roles in plant responses to different stresses [31]–[32]. However, the biological functions of PLDγs are not well understood. PLDγ1 and PLDγ2 are PIP_2_-dependent and require micromolar Ca^2+^ for activity [33], [34]. PLDγ1 and PLDγ2 are 90% identical in amino acid sequences, but they differ in the effect of PIP_2_ and Triton X-100 on their activities [34]. Here, we investigated the effect of PLD mutation on Arabidopsis Al sensitivity, and the results indicate that PLDγs negatively affect plant resistance to Al stress.

## Results

### PLDγ expression in response to Al stress and production of PLDγ-deficient mutants

**Figure 1:**
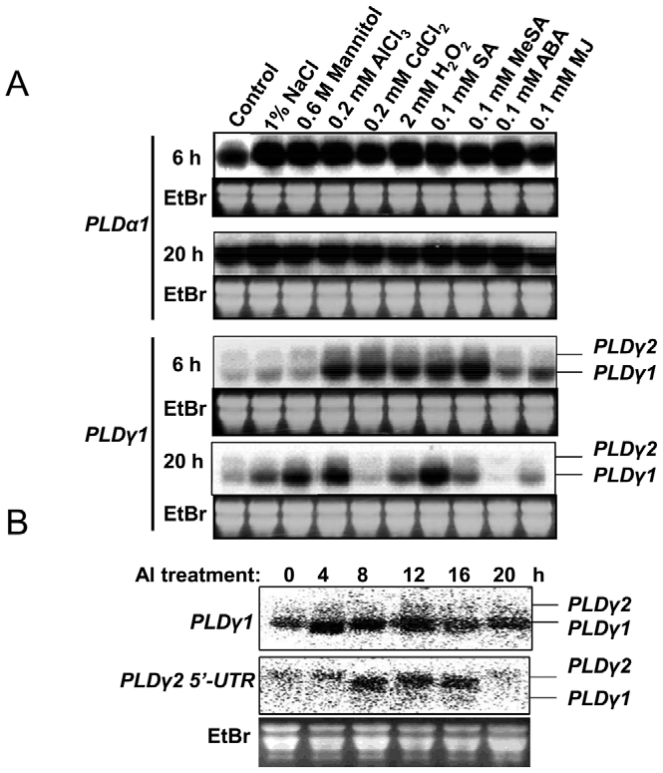
Expression of PLDs in response to various stresses. (A) RNA blotting of *PLD*α*1* and *PLD*γ transcripts in Arabidopsis leaves exposed to different stress treatments. Arabidopsis Col-0 (3–week old) leaves were detached and floated on water without or with indicated concentrations of chemicals, 1% NaCl, 0.6 M mannitol, 0.2 mM AlCl_3_, 0.2 mM CdCl_2_, 2 mM H_2_O_2_, 0.1 mM salicylic acid (SA), 0.1 mM methyl salicylate (MeSA), 0.1 mM abscisic acid (ABA), or 0.1 mM methyl jasmonate (MeJA) in a growth chamber at 22°C. Leaves were collected at the indicated times for RNA extraction and northern blotting with [α-^32^P]-labeled *PLD*α*1* or *PLD*γ*1* full-length cDNA as a probe. (B) RNA blotting of PLDγs using the coding region of *PLD*γ*1* (*upper panel*) or *PLD*γ*2-*specific 5′-UTR (*lower panel*) under Al stress. Two week-old wild-type seedlings grown in ½ MS medium were treated with 100 µM AlCl_3_, and roots of seedlings were collected at the indicated time for RNA extraction. [α-^32^P]-Labeled *PLD*γ*1* cDNA or *PLD*γ*2*- specific 5′-UTR was used as a probe for northern blotting. EtBr-stained ribosomal RNA was used as a loading control.

To obtain clues about the physiological functions of different PLDs, we examined the expression patterns of *PLD*s in detached leaves treated with various hormones and abiotic stresses for 3 and 20 h. The level of *PLD*γ was induced by treatment with AlCl_3_, cadmium (Cd), H_2_O_2_, salicylic acid (SA), and methyl salicylate (MeSA) at 3 h of treatment. The induction of *PLD*γs by Al-was transient and *PLD*γ expression fell close to the basal level by 20 h (Fig. 1A). At 20 h, the induction of *PLD*γmRNAalso occurred in response to NaCl, mannitol, CdCl_2_, MeSA, SA, and abscisic acid (ABA) (Fig. 1A). In contrast, the basal level of *PLD*α*1* expression was high and some increase in its expression occurred at 3 h after all treatments, but no induction of mRNA was detectable with 20-h treatments (Fig. 1A). To determine whether both of the two individual *PLD*γs were induced by Al stress, Arabidopsis seedlings were treated with 100 µM AlCl_3_ (pH 4.0) and sampled at various time points to examine *PLD*γ expression with *PLD*γ*1* cDNA and the *PLD*γ*2* 5′UTR as probes (Fig. 1B) [34]. Both *PLD*γ*1* and *PLD*γ*2* were induced transiently by Al stress; the induction peaked earlier for *PLD*γ*1* than *PLD*γ*2*, and both fell close to the basal level at 20 h. A survey of EST-based gene expression in Al-stressed rye also identified *PLD*s as responsive genes [35].

**Figure 2:**
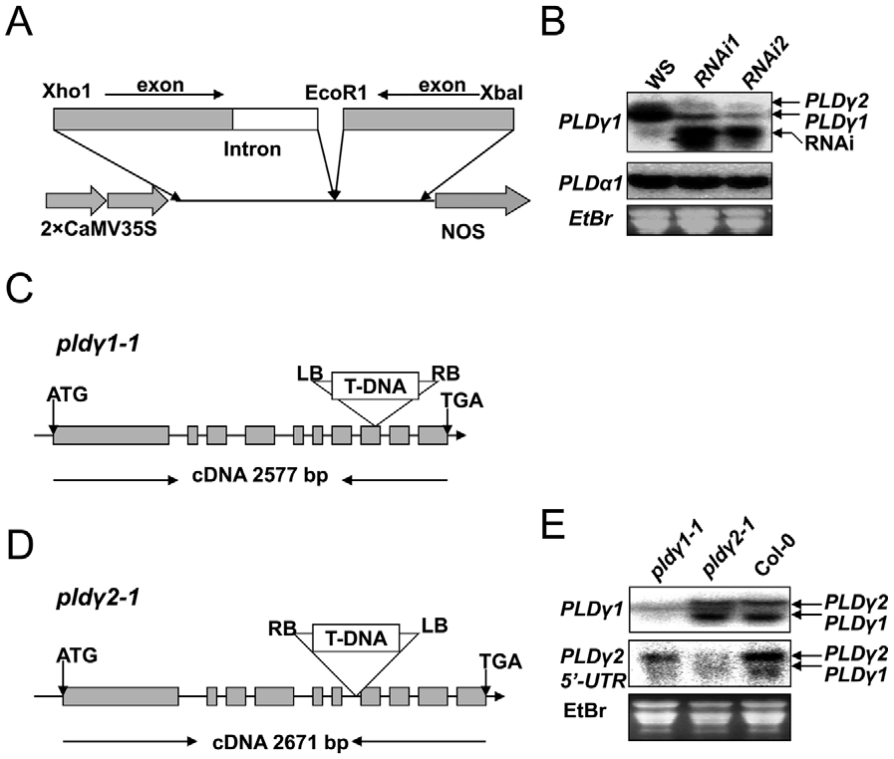
Generation of PLDγ RNAi and T-DNA insertional knockout mutants. (**A**) RNAi suppression construct. Inverted-repeats of an exon (gray boxes) and an intron (empty boxes) of *PLD*γ*1* with restriction sites were cloned in a tandem and expression was driven by double CaMV 35S promoters and terminated by an NOS terminator. (**B**) Northern blotting analysis of *PLD*γ*1* gene expression in *RNAi1* and *RNAi2* lines (*right panel*). The same RNA was probed for *PLD*α*1* expression as a control. EtBr-stained ribosomal RNA was used as a loading control. (**C**) T-DNA insertion mutant of *PLD*γ*1*. Grey boxes show exons and lines between boxes represent introns; T-DNA is inserted at the 8th exon of *PLD*γ*1* (At4g11830, 1912 bp from the start codon of the cDNA). The left border (LB), right border (RB), and direction of T-DNA (arrow in T-DNA) are shown. (**D**) T-DNA insertion mutant of *PLD*γ*2*. T-DNA is inserted at the 6th intron of *PLD*γ*2* (At4g11850, 1635 bp from the start codon of cDNA). (**E**) Northern blotting of *PLD*γ*1* and *PLD*γ*2* transcripts in mutants and Col-0 using *PLD*γ*1* cDNA and the *PLD*γ*2-*specific 5′-UTR of as probes. EtBr-stained 18S RNA is a loading control.

To characterize the biological functions of the PLDγs, we generated PLD-RNAi transgenic plants (Fig. 2). Two selected lines, PLDγ*RNAi1* and PLDγ*RNAi*2, had decreased levels of PLDγ transcripts (Fig. 2A). Quantification of the RNA blotting data indicated that the *PLD*γ mRNA levels in *PLD*γ*RNAi-1* and *PLD*γ*RNAi-*2 plants were reduced by approximately 70–85% and 90%, respectively, compared with wild-type controls (Fig. 2B).

Two T-DNA insertion mutants were isolated from Salk T-DNA insertion lines (Fig. 2C, D). Sequencing indicates that *pld*γ*1-1* contains a T-DNA insert at the 8th exon of *PLD*γ*1*, 1912 bp from the start codon (Fig. 2C). Mutant *pld*γ*2-1* has a T-DNA insert at the 6th intron of *PLD*γ*2*, 1635 bp from the start codon (Fig. 2D). Because PLDγs share more than 90% identity at the DNA level; distinguishing their transcripts in these mutants was difficult. However, *PLD*γ*2* has a longer 5-UTR than *PLD*γ*1* [34] so we used the PLDγ1 full-length cDNA and the long 5-UTR of *PLD*γ*2* cDNA as probes to detect their transcripts in *pld*γ*1-1* and *pld*γ*2-1* mutants. Under strict hybridization conditions, *pld*γ*2-1* plants did not exhibit the higher band, whereas *pld*γ*1-1* did not exhibit the lower band (Fig. 2E). The result indicates that *PLD*γ*1* and *PLD*γ*2* transcripts are absent or decreased substantially in *pld*γ*1-1* and *pld*γ*2-1* mutants, respectively.

### Altered responses of PLDγ mutants to Al stress

**Figure 3:**
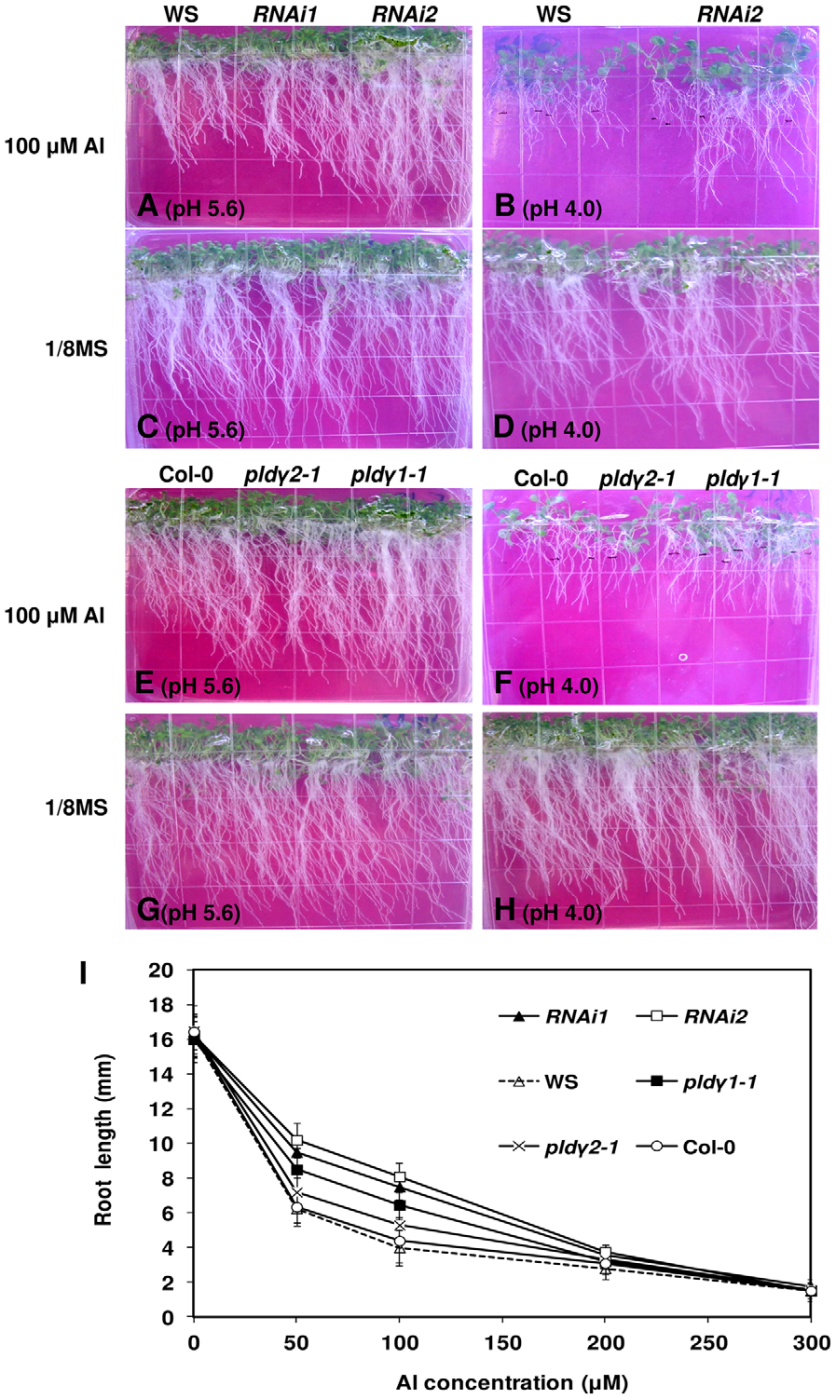
Root growth of PLDγ mutants and wild-type Arabidopsis under Al stress. (A-D) PLDγ RNAi mutants and wild-type WS in 1/8 MS medium containing 100 µM AlCl_3_ (A and B) or in 1/8 MS medium without AlCl_3_ (C and D) at two different pHs. (E–H) Col-0, *pld*γ*1-1, pld*γ*2-1* mutants on 1/8 MS plates containing 100 µM AlCl_3_ (E and F) and 1/8 MS medium without AlCl_3_ (G and H). Photos are representatives from at least three independent experiments (7 day-old seedlings post treatment).

**Figure 4:**
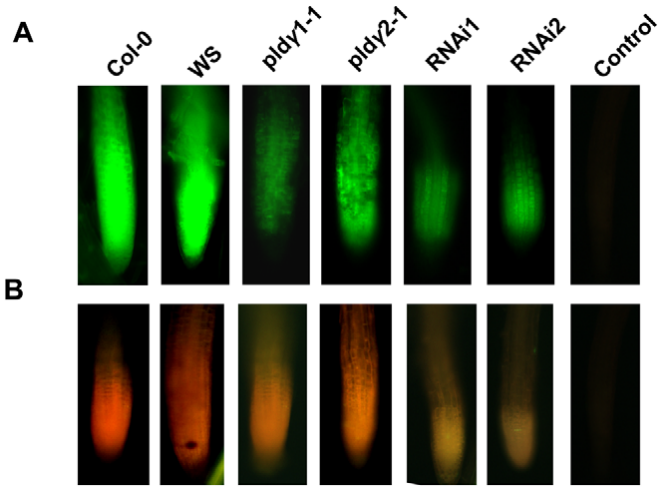
Root length and callose accumulation of PLDγ mutants and wild-type roots. (A) Quantification of root length of PLDγ mutants and wild-type seedlings at various concentrations of AlCl_3_ (pH 4.0). Four day-old seedlings were transferred to ^1^/_8_ MS containing indicated levels of AlCl_3_. Roots were measured 5 days after transfer. The values of *RNAi1* and *RNAi2*were significantly different (P<0.05) from those of WS and the values for *pld*γ*1-1* were significantly different than the values for Col-0 at 50 and 100 µM AlCl_3_. Data represent the mean ± SD (N = 50). (B) Callose staining in roots. Seven day-old seedlings were treated with 100 µM AlCl_3_ in 1/8 MS solution (pH 4.0) for 5 h and roots were stained for callose with anniline blue as described in “Materials and Methods”. Images are representatives of three experiments and more than 25 roots. Control was Col-0 roots without Al treatment; all genotypes had no staining without the Al treatment.

Root elongation of PLDγ mutants and wild-types was measured at different pHs (Fig. 3) and different concentrations of AlCl_3_ under acidic pH conditions (Fig. 4). The seedling root growth of both ecotypes, Col-0 and WS, was retarded compared with controls, when the plants were transferred to plates containing 100 µM AlCl_3_ (Fig. 3). The retardation by Al was greater at pH 4.0 than at pH 5.6 (Fig. 3). Low pH (around 4.0) is required for Al toxicity, because Al^3+^ is not chelated and precipitated at low pH. Roots of *PLD*γ RNAi and *pld*γ*1-1* seedlings at 50 and 100 µM Al lengthened significantly more than wild-type controls (P<0.01), whereas *pld*γ*2-1* did not (Fig. 4A). In addition, PLDγ RNAi mutants showed increases in the number and length of hairy roots under Al stress conditions (data not shown). The effect of PLDγ mutants on root growth under Al stress was dependent on Al concentration; the root length was similar between PLDγ mutants and wild-types in media without Al, at Al levels higher than 200 µM and media where the pH was raised to pH 5.6, where Al is not toxic (Fig. 3 and 4A).

Callose deposits on cell walls are another commonly observed phenomenon in plants in response to Al stress and have been used as a marker of Al stress intensity and damage [19], [36], [37]. By staining with aniline blue, callose production in Al-treated roots was visualized. The roots of wild-type plants of both ecotypes show a strong brown fluorescent signal, indicating increased callose accumulation. PLDγ RNAi and *pld*γ*1-1* mutants had reduced yellowish fluorescent signals compared to the wild-type controls, whereas the staining intensity of *pld*γ*2-1* was close to that of its wild-type control (Fig. 4B).

### Effect of PLDγ mutations on Al-induced organic acid secretion and Al content

**Figure 5:**
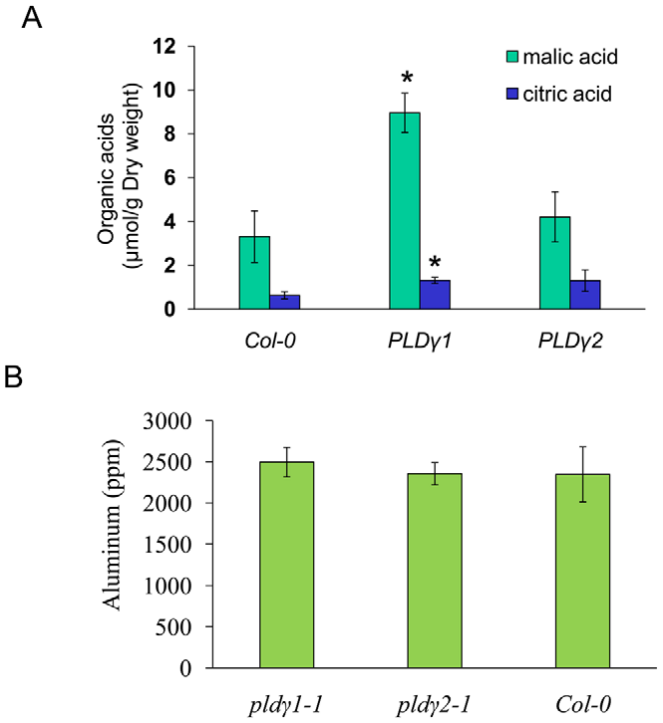
Al content in roots and organic acid released by roots of PLDγ mutants and wild-type. Wild-type, *pld*γ*1-1*, and *pld*γ*2-1* mutant seedlings were treated in 1/8 MS medium (pH 4.0) containing 50 µM AlCl_3_ for 5 hrs. Media were used for organic acid analysis with GC-MS (A) and roots were exercised and washed for ICP-MS analysis of Al content (A). Data are from three samples and presented with means ± S.D. * indicates *p*<0.05, significant difference in Student's *t* test.

Al stress also induces plasma membrane transporters to secrete organic acids that can chelate Al, and the release of citric acid and malic acid to root environments is closely associated with Al-tolerance in plants [9]–[12]. After treatment of roots of wild-type and PLDγ mutants with 50 µm AlCl_3_ for 5 h, the secretion of citric acid and malic acid into the media was measured. The roots of *pld*γ*1-1* excreted significantly higher levels of citrate and malate into the media (Fig. 5A). Al accumulation in Al-treated *pld*γ mutants and wild-type was measured in Al-treated roots by inductively coupled plasma mass spectrometry (ICP-MS). We did not detect significant differences in root Al accumulation between *pld*γ*1-1, pld*γ*2-1,* and Col-0 roots treated with 50 µM Al for 5 h (Fig. 5B).

### Altered oxidative stress in PLDγ mutants in response to Al stress

**Figure 6:**
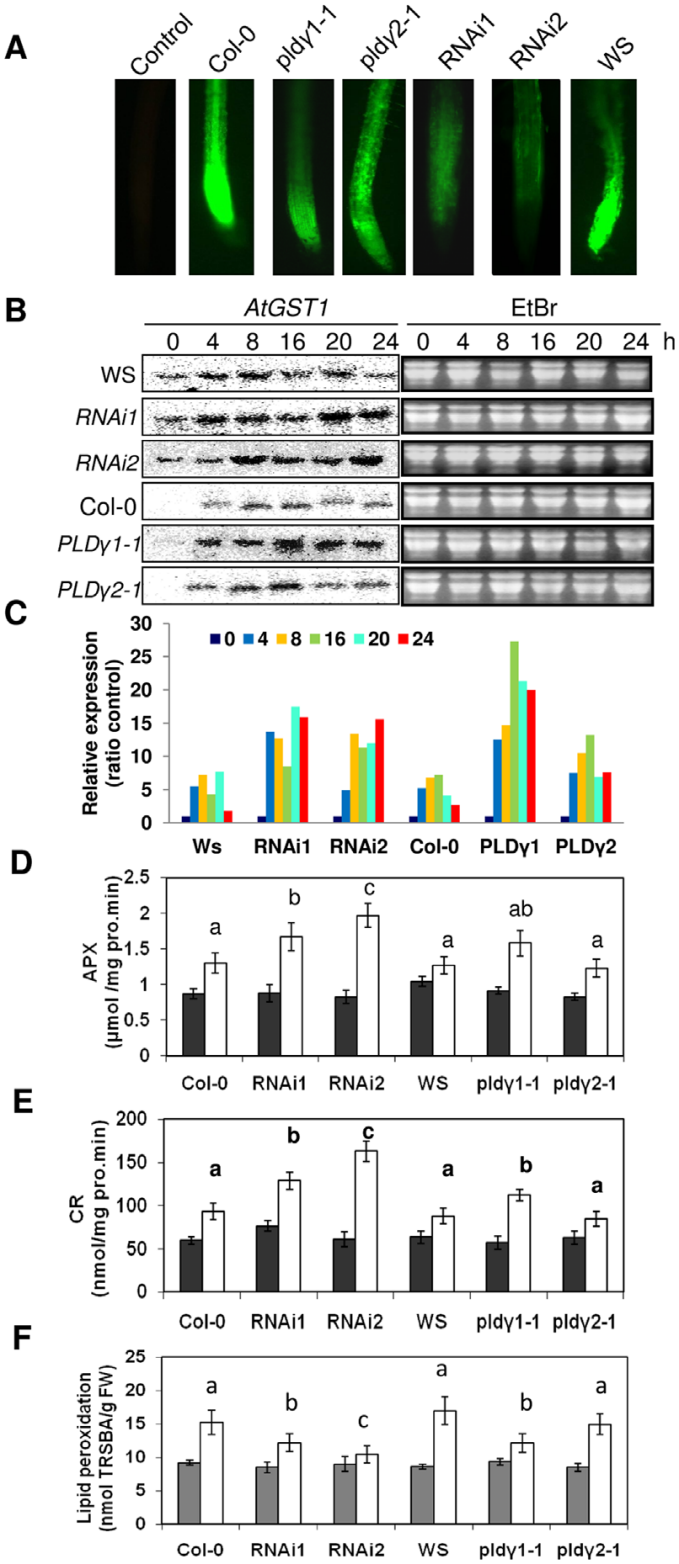
Changes in oxidative stress and antioxidant activity. Seven day-old PLDγ mutant and wild type seedlings were treated (white bars) or not treated (black bars) with 100 µM AlCl_3_ in 1/8 MS solution (pH 4.0) for 5 h or indicated time (for northern blotting). Then roots were harvested for imaging, northern blotting, enzyme activity assay, and lipid peroxidation measurments. (A) ROS generation under Al stress. Roots were stained with 2′,7′-DCFDA for ROS imaging as described in “Materials and Methods”. Images are representatives of three experiments and more than 25 roots. (B) GST expression in plants with altered *PLD*γ expression under Al stress. RNA extracted from Al-stressed seedling roots for different time was used for northern blotting with [α-^32^P]-labeled full-length *AtGST1* cDNA as the probe. EtBr-stained ribosomal RNA was used as loading controls. (C) Quantification of *AtGST1* expression. Quantification was based on band intensity and expressed as ratio to the controls (0 h of treatment). (D) APX and (E) GR activity. Al-treated or control roots were sampled for the enzyme activity assays as described in “Materials and Methods”. (F) Lipid peroxidation. Roots were sampled for assay as described in “Materials and Methods”. Data represent means ± SD (N = 3) from three independent experiments. Label ‘a’ above the bar indicates that the value of the Al-treated is significantly different from the untreated sample of the same genotype at P<0.05. Labels ‘b’ or ‘c’ above the bar indicate that the mutant values are significantly different from wild type with the same treatment at P<0.05.

Al stress induces oxidative stress in plant roots, and increases in oxidative stress are regarded as one of the causes of root damage by Al [6], [18], [20], [37]. The reactive oxygen species (ROS)-detecting agent, 6-carboxy-2,7-dichlorodihydrofluorescein diacetate (2,7-DCFDA), was used to examine whether there is any alteration in ROS production in PLDγ mutants compared to wild-type plants under Al stress. Roots of seedlings grown in 50 and 100 µM AlCl_3_ displayed strong 2,7-DCFDA fluorescence (Fig. 6A). The signal was much stronger in roots than in other tissues (data not shown), which is consistent with the observation that the Al-induced formation of ROS occurs first in Arabidopsis roots [6], [18], [20]. *RNAi1, RNAi2*, and *pld*γ*1-1* exhibited much weaker fluorescent staining than wild-type, and the lower signal intensity occurred in both root cells and throughout the whole root apex in seedlings grown in 100 µM AlCl_3_. The fluorescent staining of *pld*γ*2-1* was stronger than the other mutants but slightly weaker than wild-type Co1-0 (Fig. 6A). Glutathione *S*-transferase (*GST*) is up-regulated in oxidative stress caused by many adverse environments [38]. At*GST1* expression increased upon Al stress; At*GST1* expression in both *pld*γ*1-1* and *RNAi* mutants was higher than their respective wild type controls and *pld*γ*2-1* (Fig. 6B and C).

To examine the potentially altered balances in oxidative stress and antioxidant systems, the ascorbate-glutathione antioxidant cycle system was examined by assaying the activities of ascorbate peroxidase (APX) and glutathione reductase (GR) in PLD mutants and wild-type plants that were exposed to Al for 5 h. *RNAi* mutants and *pld*γ*1-1* plants had higher APX and GR enzymatic activities than wild-types, while activities of the two enzymes in *pld*γ*2-1* were comparable to those in wild-type plants (Fig. 6D and E). In addition, PLDγ RNAi and *PLD*γ*1*mutants had significantly lower levels of lipid peroxidation, as assayed by the thiobarbituric acid reaction (TBARS), whereas the lipid peroxidation value of *pld*γ*2-1* was similar to that of wild-type (Fig. 6F). Of all the genotypes, *PLD*γ*RNAi2* had the highest APX and GR activities and lowest lipid peroxidation value (Fig. 6).

### Membrane lipid changes upon Al stress

**Figure 7:**
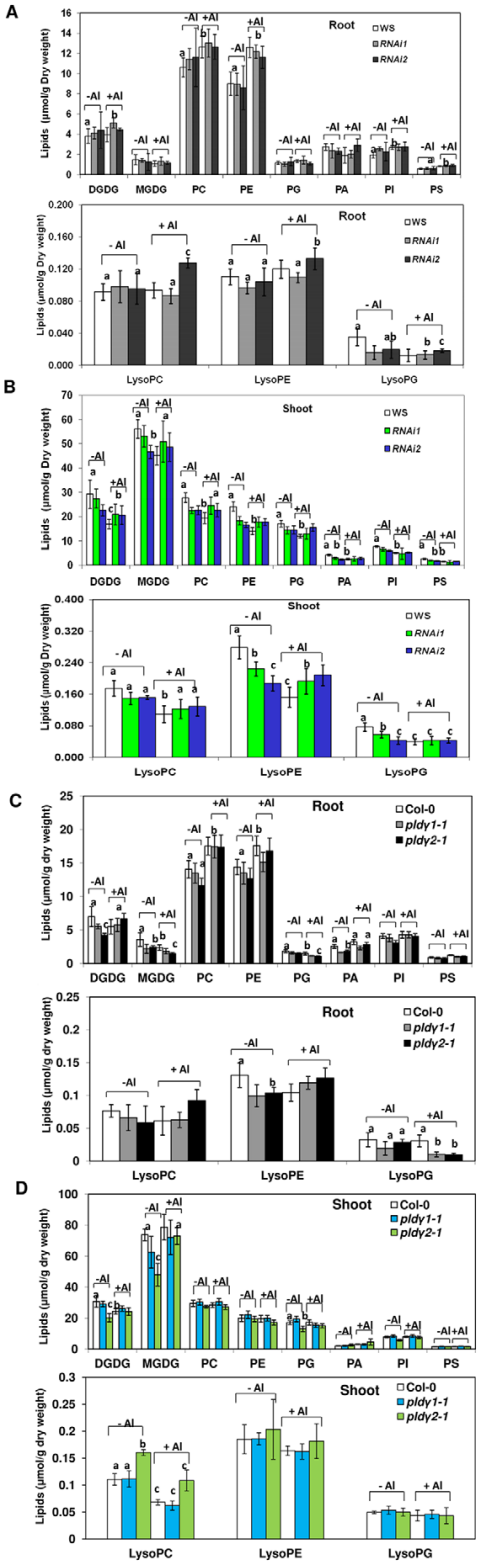
Lipid changes as affected by Al stress and PLDγ mutations. Seven day-old PLDγ mutant and wild type seedlings were treated with (+Al) or without 100 µM AlCl_3_ (-Al) in 1/8 MS solution (pH 4.0) for 2 days. Roots and shoots (rosette leaves and stems) were harvested separately for lipid extraction as described in “Material and Methods”. Data represent means ± SD (N = 5). (A) and (B) Lipid profiles of PLDγ RNAi mutants and wild-type (WS) seedlings in roots and shoots, respectively. (C) Lipid profiles of PLDγ1-KO and PLDγ2-KO and wild-type (Col-0) seedlings in roots and shoots, respectively. (Label ‘a’ above the bar indicates that the value of the Al-treated is significantly different from that of non-treated control at P<0.05. Label ‘b’ above the bar indicates that the mutant values are significantly different from wild type at P<0.05. Label ‘c’ above the bar indicates that the mutant values are significantly different from wild type and non-Al treatment control at P<0.05.

To examine lipid changes occurring as a result of Al stress and PLDγ–deficiencies, seedlings of PLD mutants and corresponding wild-types were transferred to 100 µM AlCl_3_ for two days. Roots and shoots (including stems and rosette leaves) under the Al-stressed and normal conditions were collected separately for phospholipid and glycolipid analysis by an ESI-MS/MS method described previously [39]. Compared with untreated roots, the levels of several phospholipids, PC, PE, PI, and PS, were increased in roots of both WS (Fig. 7A) and Col-0 seedlings under Al treatment (Fig. 7C). Changes in response to Al treatment of plastidic lipids, DGDG, MGDG, and PG, were minor (Fig. 7A and C), but a significant drop in MGDG levels was observed in the roots of Col-0 plant under Al stress (Fig. 7C). In shoots of wild-type plants, the most consistent change due to Al treatment, occurring in both ecotypes, were decreases in the levels of DGDG and lysoPC (Fig. 7B and D).

Without Al stress, PA was lower in *pld*γ*-1 and pld*γ*2-1* roots and RNAi lines leaves (Fig. 7A and B), suggesting that PLDγ*1* and *PLD*γ*2* play a role in the basal accumulation of PA. With Al stress, the level of PA tended to increase in Col-0 and the mutants *pld*γ*1-1* and *pld*γ*2-1*, and the percentage of increase was greater in the mutants, particularly in *pld*γ*2-1*. The results suggest that these PLDs are not responsible for Al-induced PA increase. This observation is consistent with an earlier finding that Al inhibits phosphoinositide-dependent PLD activity in vitro [27].

**Figure 8:**
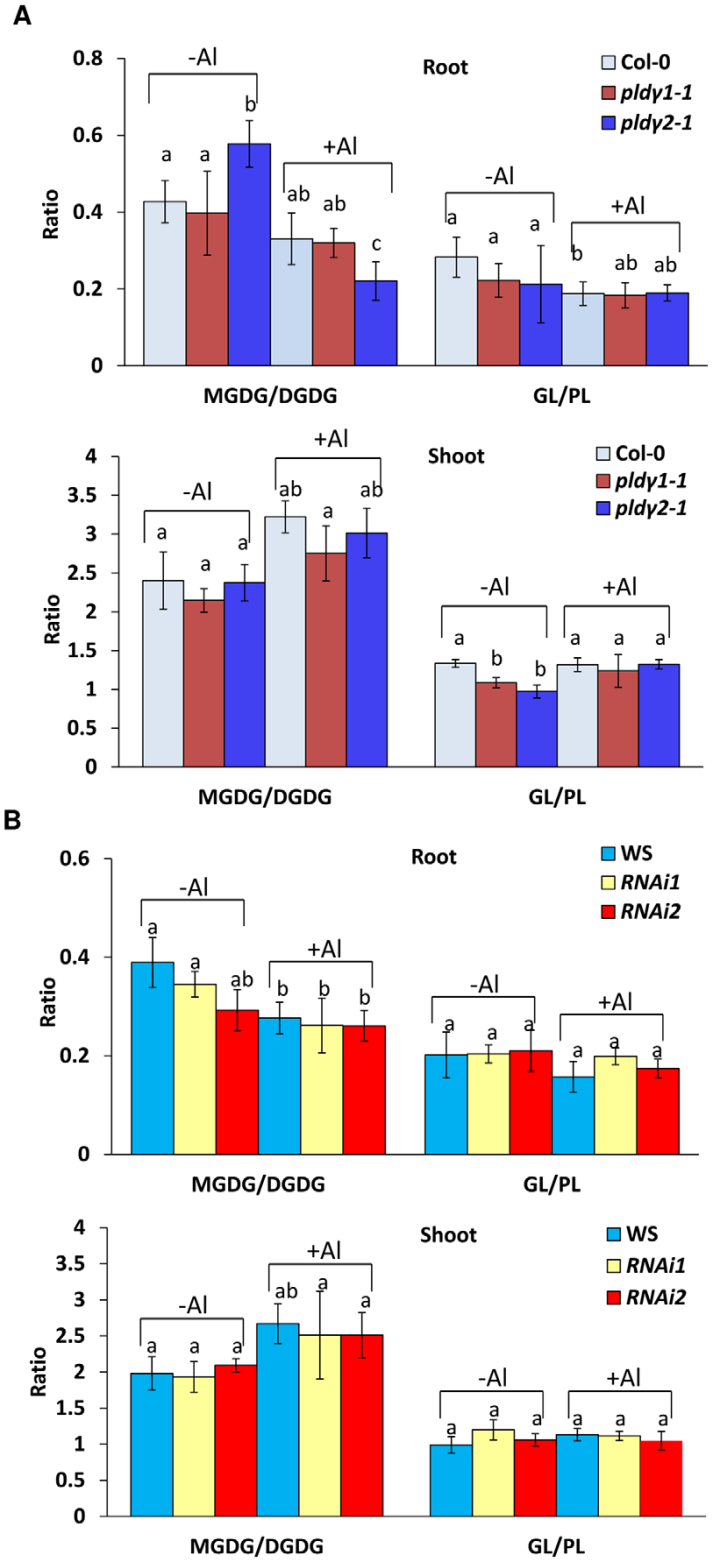
Relative changes of glycolipids and phospholipids as affected by Al in roots and shoots. (A) Ratio of total MGDG/DGDG and glycolipids (GL, sum of total DGDG and MGDG) to phospholipids (PL, sum of total all phospholipids and lysophospholipids) from roots and shoots of PLDγ RNAi mutants and WS seedlings. (B) Ratio of MGDG/DGDG and GL/PL from roots and shoots of Col-0, *pld*γ*1-1,* and *pld*γ*2-1* seedlings. The ratios were calculated based on the data in Figure 6. Seven-day-old PLDγ mutant and wild type seedlings were treated with 100 µM AlCl_3_ (+Al) or without Al (-Al) at pH 4.0 for 2 days. Different letters above the bar indicate sample groups with significant differences (P<0.05) from each other, whereas the same letters indicate no significant difference from each other.

PLDγ mutants show fewer significant changes in all major phospholipids and glycolipids than wild-types under Al stress (Fig. 6B and D). All mutants and wild type controls showed a decrease in MGDG/DGDG ratio after Al treatment in roots (Fig. 8). On the contrary, the MGDG/DGDG ratio in shoots was increased in all wild-types and mutants under Al stress. The results suggest that lipids in roots and shoots change differently in response to Al stress.

## Discussion

The study shows that the suppression of PLDγs renders Arabidopsis seedlings more tolerant to Al stress. Analysis of the single gene knockouts suggests that PLDγ1 is the gene responsible for most of the observed Al tolerance in the PLDγ RNAi lines. This is not surprising given that *PLD*γ*1* is expressed in the roots where most of the Al toxicity is observed while *PLD*γ*2* predominantly expressed in the inflorescence [34]. This suggests that Al stress is similar to other abiotic and biotic stress conditions, such as drought, hyperosmotic stress, and phosphorous starvation where PLDs have been implicated in mediating dynamic changes of membrane phospholipids and glycolipids [31], [32], [40].

The increased Al tolerance in PLDγ RNAi and *pld*γ*1-1* plants is accompanied by the decreased levels of ROS, lipid peroxidation, and callose deposition in roots, and by the increases in the expression of protective *GST* and activities of redox enzymes APX and GR. ROS generation and oxidative stress are known to be increased by Al treatment of Arabidopsis [4], [6], [18], [20], [37] and responses such as increasing *GST* expression and activating redox enzymes are used to protect membrane lipids from peroxidation [4], [6], [18], [20], [38]. Al stress increases ROS production in all genotypes, but Al-resistant PLDγ mutants accumulate less ROS than wild-type roots, suggesting that the ROS level is associated with cell damage. ROS is a double-edged sword: at low levels and earlier stages of stress, ROS may trigger signaling and defense responses such as increasing callose production and rigidification of the cell wall [6], [18]–[20], [27], [37], [38]. However, a large increase in ROS can result in and/or be indicative of cell damage. Studies have suggested that mitochondrial dysfunction is an Al toxicity mechanism due to increased mitochondria membrane permeability, excess ROS generation, and redox signals [4], [6], [20], [38], [41]. Subcellular localization results indicate that PLDγ1 is primarily associated with membrane fractions, including mitochondrial and plasma membranes and nucleus [42]. Since the expression of *PLD*γs is induced by H_2_O_2_ and Al stresses, and PLDγ1 can also be phosphorylated, which might regulate PLDγ1 activity in lipid metabolism [43], it would be possible that PLDγs are involved in signal transduction and mitochondrial or plasma membrane lipid hydrolysis under Al stress. Thus, PLDγ might be required for certain Al- activated stress responses such as ROS generation, redox status change, and lipid peroxidation. PLDγ1-deficient mutants thus have smaller changes in lipid compositions or oxidative stress damage under Al stress since the suppression of PLDγs may partially block ROS production and attenuate Al-induced damage. Furthermore, PLDγ RNAi and *pld*γ*1-1* mutants displayed higher ROS-scavenging enzyme activity. APX and GR are responsible for regeneration of two important antioxidants, ascorbate and glutathione [41]. The higher activities of these enzymes in PLDγ RNAi and *pld*γ*1-1* mutants are consistent with the decreased lipid peroxidation and membrane damage.

PLDγs could affect membrane lipid metabolism under Al stress by affecting lipid degradation and/or by lipid remodeling, such as replacement of phospholipids with glycolipids under phosphorous starvation [40], [44]. Degradation is primarily detrimental and leads to fatty acid release and oxidation. The treatment of plants with Al and other metals causes changes in the composition and levels of phospholipids and glycolipids [4], [5], [15], [28], [45]–[48]. Lipid composition affects the membrane integrity, fluidity, and biological functions of proteins intrinsic to and associated with membranes. The biological significance of the lipid changes is not well understood and enzymes catalyzing the membrane lipid changes in response to metal stresses are not defined. Our results suggest that suppression of PLDγs decreased Al-induced lipid changes and lipid peroxidation. An increase in lipid peroxidation after Al treatments was associated with a decrease in polyunsaturated fatty acids in sorghum, and the decrease on polyunsaturated fatty acids was more severe in an Al-sensitive sorghum cultivar than in an Al-tolerant one [4], [5], [15]. A similar association between decreased lipid peroxidation and enhanced Al tolerance was also observed in sorghum and rice cultivars, as well as transgenic Arabidopsis plants [18], [19], [49], [50]. The results suggest that the suppression of PLDγs decreases lipid hydrolysis and partially blocks Al-induced membrane damage.

The lipid compositional results indicate that plant roots and shoots respond differently to Al stress. MGDG/DGDG ratio was decreased in roots, but increased in shoots in mutants and wild types after Al treatment. Earlier studies indicated that Al increased MGDG/DGDG ratio in membranes from maize roots [15] and from roots of the Al-tolerant wheat cultivar [5]. Thus, our results in roots are different from those reported in maize and wheat. It is not clear whether it is due to a difference between monocots and dicots. In addition, PLDγ RNAi mutants had a lower root MGDG/DGDG ratio than WS control under normal growth conditions. In *pld*γ*1-1, pld*γ*2-1,* and Col-0 seedlings, MGDG/DGDG ratio in roots all decreased in response to Al stress, and *pld*γ*2-1* had a larger decrease than did *pld*γ*1-1* and Col-0. Thus, the present data showing a drop in MGDG/DGDG ratio in all genotypes and no consistent trend for difference in amount of change between wild-types and mutants, do not support a positive association of increased Al tolerance with increased MGDG/DGDG ratio in roots. In contrast, our data suggest that Al-resistant lines are likely to maintain a relatively constant MGDG/DGDG ratio in leaves.

Characterization of various Arabidopsis Al-sensitive mutants suggests that tolerance of Arabidopsis to Al toxicity involves more than one mechanism, and the mechanism may be unique to specific plant species [51]. Secretion of organic acids, such as citric acid and malic acid [12], [52], increase in the rhizosphere pH [53], relocation of Al to less sensitive tissues [3], and improved integrity and function of membranes [7] are all potential mechanisms for Al tolerance. An Al tolerance phenotype may result from one or more mechanisms. The present results indicate that increased changes in major membrane glycerolipids are positively associated with Al sensitivity and that PLDγs are involved in Al-induced cell damage. The lipid changes in response to Al are distinctively different in roots and leaves. PLDγs are involved in the Al resistance by affecting Al-evoked lipid hydrolysis that results in alterations in oxidative stress. The generation of double and triple mutants of PLDγs, which has been difficult because the genes are linked in tandem [54], will aid further elucidation of the role of PLDs in plant response to Al toxicity.

## Materials and Methods

### Plant Materials and Mutant Isolation

*Arabidopsis thaliana PLD*γ*1* (At4g11850) and *PLD*γ*2* (At4g11830) were cloned as previously described [33], [34]. T-DNA insertion knockouts for *PLD*γ*1* and *PLD*γ*2* were isolated from Salk lines SALK_113873 (*PLD*γ*1*) and SALK_014510 (*PLD*γ*2*) obtained from ABRC (Columbia OH), by using gene specific primers, 5′-TCATATGGTGAGGTTTTCTTGTAG-3′ for *PLD*γ*1* or 5′-ATGTCAATGGGAGGAGGG-3′ (for *PLD*γ*2*) with a T-DNA left border primer. These two homozygous mutants show a co-segregation between their PLDγ deletions with kanamycin resistance, suggesting that these mutants have a single T-DNA insertion.

### PLDγ RNAi Construct

The sense cDNA exon (362 bp) and the following intron (283 bp) were amplified by PCR with forward primer GIR51: 5′-CCGCTCGAGTGGTAATGAGTGTGTAGGAGTTC-3′ (*Xho* I underlined) and reverse primer GIR31: 5′-CCGGAATTCTTGCTACAACAAAACAAAAGCTT-3′ (*EcoR* I underlined). Antisense cDNA (362 bp) was amplified with forward primer GIR52: 5′-CCGGAATTCTTTTGAATCCCAGAAGACTC-3′ (*EcoR* I underlined) and reverse primer GIR32: 5′-CTAGTCTAGATGGTAATGAGTGTGTAGGAGTTC-3′ (*Xba* I underlined). The two PCR products were digested with *EcoR* I and then ligated into a fragment of 1007 bp containing two inverted cDNA repeats separated by an intron. This fragment was further digested with *Xho*I and *Xba*I restriction enzymes, and the resulting fragment was purified and then ligated into pKYLX71-35S^2^ binary vector at *Xho*I and *Xba*I sites. The resulting RNAi construct was confirmed by sequencing and used to transform *Agrobacterium tumefaciens* strain GV301 and then Arabidopsis plants, using the floral dip method. F1 to F3 progeny were screened on kanamycin plates and using PCR. Two homozygous lines, *PLD*γ*RNAi1* and *PLD*γ*RNAi2*, were obtained with dramatically decreased PLDγ transcripts as indicated by northern blotting.

### Root Elongation Assay

All seeds from *PLD*γ*1* and *PLD*γ*2* T-DNA knockouts, PLDγ RNAi mutants, or their wild-type controls (WS for RNAi mutants and Columbia ecotype Col-0 for T-DNA knockouts) were collected at the same stage for the root elongation test. Seeds were surface-sterilized and germinated on ½ Murashige and Skoog (MS) plates containing 1% sucrose and 0.8% agar. Four to seven day-old seedlings were transferred to ^1^/_8_ MS containing 50, 75, 100, 150, 200, 250, and 300 µM of AlCl_3_, 1% sucrose, and 0.8% phytagar (pH 5.6 or pH 4.0, pH was adjusted with 5 mM MES). Plates were vertically cultivated under cold fluorescent light with a 16 h/8 h light period at 23°C. The newly elongated roots were measured.

### RNA Blotting

Total RNA was isolated from roots or leaves of Arabidopsis plants with a cetyltrimethylammonium bromide extraction method [55]. Equal amounts of total RNA (10 µg) were separated by 1% formaldehyde agarose denaturing gel electrophoresis and transferred to nylon membranes. The entire coding regions of *PLD*γ*1* and *PLD*γ*2* 5′-UTR, or full-length AtGST1 cDNA were used as hybridization probes. The DNA probes were labeled with [α-^32^P] dCTP by random priming. The hybridization, washing, and visualization were performed as described previously [35].

### Microscopic Observations

Cell death caused by Al treatments was monitored by DAPI staining. After treatment of 7 day-old seedlings with 100 µM Al^3+^ in ^1^/_8_ MS medium for 5–8 h, roots from different plants were fixed with ethanol and stained with DAPI for 15 min. ROS generation in roots was observed by using 2,7-DCFDA as a detecting agent. Seedlings were treated as above for 5 h and then were put into a fresh PBS buffer; 5 µg/ml of 2,7-DCFDA were added to stain the ROS. Roots were imaged under a fluorescence microscope (Nikon Eclipse 800, Japan) with excitation at 488 nm and emission at 540 nm to detect green fluorescence intensity.

Callose production in Al-treated roots was visualized by staining with aniline blue as described by [3]. The root region of 7 day-old seedlings was exposed to ^1^/_8_ MS medium containing 100 µM Al for 5 h, fixed with formaldehyde under vacuum, and then stained with 0.1% (w/v) aniline blue in 0.1 M K_3_PO_4_ (pH 9.0). Callose was imaged with a fluorescence microscope (Nikon Eclipse 800, Japan) with excitation at 365 nm and emission at 515 nm.

### Measurements of Al accumulation and organic acid secretion

Accumulation of aluminum in roots and secretion of organic acids into medium upon AlCl_3_ treatment were determined by growing seedlings on ½ MS medium plates oriented vertically. After 7 days, the plants were transferred to 1/8 MS liquid medium (pH 4.0) for adaptation for 2 h followed by 50 µM AlCl_3_ Al-treatment for 5 h. After treatment, the media were collected for organic acid analysis by GC-MS. The roots were rinsed in fresh 1/8 MS medium without AlCl_3_ for 5 min (medium changed twice), the roots were roots cut off and put at 90°C overnight; completely dried roots were used for ICP-MS (inductively coupled argon plasma mass spectrometer) analysis. Al was analyzed according to the method described previously [56].

To measure organic acids secreted by plants, medium (50 mL) was passed through a cation exchange column (15 mm×5 cm) filled with Bio-Rad AG50W-X8 resin (Bio-Rad, Richmond, CA). The eluent was passed through an anion exchange column (15 mm×8 cm) filled with Dowex-1 1×2−100 resin. Organic acids retained on the anion-exchange resin were eluted with 40 mL of 2 M HCl. 2 mL of the eluent were dried completely by vacuum centrifugation, and the organic acids were methylated using 0.5 mL of 1.25 M methanolic HCl for 8 h at 50°C. Samples was dried under nitrogen and reconstituted in 100 µL of pyridine. Organic acids were quantified using an Agilent 6890 GC coupled to a 5973 MSD. One µL was injected in a split/splitless injector at 280°C at a split ratio of 15∶1. Separation was achieved on DB-5 MS column (J&W Scientific, 60 m, 0.25 mm i.d., and 0.25 µm film). Helium was the carrier gas at a constant flow of 1 mL/min. The GC temperature program was 80°C for 2 min then ramped to 315°C at 5°C/min was held at 315°C for 12 min. The transfer line to the mass spectrometer was set to 280°C and the MS source was set to 230°C. Mass spectra were scanned from *m/z* 50–550 at an acquisition rate of 2 spectra/s. Malic and citric acid were quantified using authentic standard that was methylated and analyzed using the same procedure.

### Enzyme Activity and Lipid Peroxidation Assay

Whole roots of Al-treated plants (100 µM Al treatment for 5 h) were frozen in liquid nitrogen. Enzyme extraction, activity assay, and lipid peroxidation determination by measuring the level of thiobarbituric acid reactive substances (TBARS) were conducted as previously described [57]. APX activity was expressed as µmol ascorbate oxidized/mg protein per min; GR activity was expressed as nmol NADPH oxidized/mg protein min, and lipid peroxidation was expressed as nmol TBARS/g fresh weight (FW).

### **Lipid Extraction and ESI-MS/MS Analysis**

Wild-type, PLDγ knockouts (*pld*γ*1-1, pld*γ*2-1*), and RNAi mutant plants were germinated and grown on ^1^/_8_ MS plates for 6–7 days in a growth chamber and then transferred to ^1^/_8_ MS medium (pH 4.0) containing 100 µM AlCl_3_ for 2 days. Roots and shoots (leaves and stems) from the seedlings were collected and immediately immersed in isopropanol (preheated to 75°C) to inactivate lipolytic enzymes. Lipid extraction and ESI-MS/MS analysis of glycerolipids was performed as previously described [39]. Five replicates of each treatment for each wild type or mutant were processed and analyzed.

### Statistical Analysis

The *Q*-test for replicates of lipid data was performed. A difference between two groups of data is considered significant when p<0.05 in the Student's *t*- test.
